# Environmental study and stress-related biomarkers modifications in a crew during analog astronaut mission EMMPOL 6

**DOI:** 10.1007/s00421-024-05575-3

**Published:** 2024-09-25

**Authors:** T. A. Giacon, Simona Mrakic-Sposta, G. Bosco, A. Vezzoli, Cinzia Dellanoce, M. Campisi, M. Narici, M. Paganini, B. Foing, A. Kołodziejczyk, M. Martinelli, S. Pavanello

**Affiliations:** 1 Department of Biomedical Sciences, University of Padova, Via Marzolo 3, 35131 Padua, Italy; 2 Institute of Clinical Physiology, National Research Council (IFC-CNR), Piazza dell’Ospedale Maggiore, 3, 20162 Milan, Italy; 3 Occupational Medicine, Department of Cardio-Thoraco-Vascular Sciences and Public Health, University of Padova, Via Giustiniani 2, 35128 Padua, Italy; 4 LUNEX EuroMoonMars, and EuroSpaceHub Academy, Leiden Observatory, Leiden, Netherlands; 5 Space Technology Centre, AGH University of Science and Technology, Kraków, Poland; 6 Analog Astronaut Training Centre, Kraków, Poland; 7 Institute of Science and Information Technologies “Alessandro Faedo”, National Research Council (ISTI-CNR), Via G. Moruzzi 1, 56124 Pisa, Italy; 8 University Center for Space Studies and Activities “Giuseppe Colombo”-CISAS, University of Padua, Padua, Italy; 9 University Hospital of Padova, Padua, Italy

**Keywords:** Non-invasive methods, Oxy-inflammation, Cortisol, Space exploration, Confinement

## Abstract

**Purpose:**

Human presence in space is increasingly frequent, but we must not forget that it is a hostile environment. We aimed to study the characteristics of experimental scenarios, to obtain data on human response to isolation, disruption of circadian rhythm and high levels of psychophysical stress.

**Methods:**

In these experiments, we evaluated stress response in five young healthy subjects inside an earth-based moon-settlement-like habitat during a 1-week long analog astronaut mission. Wearable devices were used to monitor daily step count of the subjects, physical activity, heart rate during physical exercise and at rest, and sleep parameters. From saliva and urine samples collected every day at awakening, we studied oxy-inflammation biomarkers and hormones (stress and appetite) were studied too.

**Results:**

At the end of the week, all subjects revealed an increase in oxidative stress and cortisol levels but no inflammation biomarkers variations, in conjunction with increasing time/daily exercise. Furthermore, a significant decrease in hours of sleep/day, sleep quality, and REM phase of sleep was recorded and correlated with the increase of reactive oxygen species.

**Conclusion:**

Oxidative stress increased in a short period of time and may be attributed to the influence of psychological stress during confinement, as well as increased exercise and decreased amount of sleep. On a long-term basis, this could impact performance.

## Introduction

Human exploration of space is now returning to gain importance, pushed by scientific curiosity or due to overpopulation, climate change, and lack of resources on Earth (Osborne et al. 2022; Carbajales-Dale and Murphy 2023). Nevertheless, long term space exploration imposes great challenges to human life, in particular, because of hypoxia, dysbarism, thermal support, acceleration, microgravity and high radiation levels which represent major threats to astronauts’ health (Criscuolo et al. 2020). In fact, space flight induced pathophysiological adaptive changes on the body of highly selected, well-trained, and healthy individuals (astronauts and cosmonauts) akin to an acceleration of their aging processes and to the development of some diseases. Both macroscopic (cardiovascular, musculoskeletal, and respiratory) and microscopic (molecular, endocrine and immunological) processes lead to an impairment in work ability and threaten human health (Demontis et al. 2017).

The loss of the functional capacity of the human body during space flights is much faster than on Earth. Both aging and living in space induce decline, not just of a single system, but of almost every physiological mechanism (Vernikos and Schneider 2010).

While some effects derived exclusively from space environment’s characteristics, such as microgravity and radiation, and could be studied only on astronauts, other factors, such as confinement, social stress and self-sufficiency can be easily reproduced on Earth (Cromwell et al. 2021). In fact, Earth-based analog astronaut trainings are gaining popularity and represent a perfect setting for human research and the development of useful skills and team dynamics for future astronauts (Bell et al. 2019). These trainings often took place in extreme environments, such as caves or underwater, but also appositely designed settings that resemble space infrastructures are often employed (NASA Analog Missions). Those environments are specially designed to recreate a spacecraft-like environment and to train future space crews to work, live and overcome difficulties together in simulations (Bell et al. 2019). Confinement in small spaces, loss of circadian rhythms due to shift work and natural light cycle alteration (Tahara et al. 2017; Guo et al. 2020), self-sufficiency and isolation (Jacubowski et al. 2015; Weber et al. 2019), multiculturality (Pasca et al. 2011; Jensen and Oldenburg 2020), high workload and psychophysical effort (Powers and Radak 2016) are the main stressors related to these missions and are known to increase oxidative and endocrine stress response. Stress also causes changes in daily food intake habits. In fact, some people ignore their hunger cues and refrain from eating even for long periods, while for others, stress turns them into emotional eaters who mindlessly munch (Yau and Potenza 2013). Confinement associated with stress, for example during the early months of the coronavirus disease 2019 (COVID-19) pandemic, led to changes in eating behavior (Żurek et al. 2024).

We previously demonstrated that highly stressing activities in confined environments such as saturation diving (Mrakic-Sposta et al. 2020) and offshore ocean sailing (Giacon et al. 2022) induce an increase in oxidative stress (Muid et al. 2019). People exposed to those environments endure some stressors that could be compared with those of astronauts, working as perfect experimental analogs.

The study aimed to examine how analog astronauts’ bodies respond to simulated space missions in a moon settlement-like habitat, focusing on the effects of isolation, confinement, altered circadian rhythms, and stressors on oxidative stress, inflammation, and hormones. Non-invasive measures on urine and saliva samples were used to understand how these factors influence physiological changes, potentially impacting long-duration space missions. By mimicking space conditions through analog astronaut training, the research aimed to uncover insights into oxidative stress, inflammation, and hormones levels such as cortisol, leptin, and Insulin-like growth factor-1 (IGF-1). This study deepened our understanding of challenges astronauts might face during extended space travel, shedding light on health decline mechanisms in space. Additionally, the study explored stress’s impact on eating behavior through appetite-related hormones, revealing how confinement and isolation might affect crew members’ eating habits. In sum, the study advanced our knowledge of physiological changes during prolonged space missions, offering potential strategies to address health risks and enhance future space exploration conditions.

## Materials and methods

### Selections of participants and study design

Five young analog astronauts (4 males and 1 female), whose characteristics are reported in Table 1, were recruited among the sixth Euro Moon Mars Poland analog astronaut mission (EMMPOL 6) in the Analog Astronaut Training Center (AATC Poland) as a part of the Euro Moon Mars projects by International Lunar Exploration Working Group (ILEWG).

**Table 1:** Anthropometric parameters collected from the subjects

	Mean ± standard deviation
Age (years)	22.4 ± 1.8
Height (cm)	172.0 ± 7.4
Body mass initial (kg)	65.9 ± 5.6
Body mass final (kg)	65.8 ± 5.8
BMI initial	22.4 ± 2.6
BMI final	22.3 ± 2.7

*BMI* body mass index

The subjects that voluntarily took part in this mission, were asked to stay for one week inside an Earth-based moon-settlement-like habitat. The habitat was composed of two dining/working rooms, one bedroom, one bathroom and a small gym. The total surface of the habitat was about 57 square meters. The habitat had no sources of natural light or ventilation, therefore, for the whole duration of the mission the subjects were always exposed to artificial light, even during the sleep time, and poor air quality with increased levels of CO_2_. Each subject had a role inside the mission: commander, vice-commander, crew medical officer, data officer and communication officer and were asked to perform and complete a personal experiment during the week. Other tasks included permanent experiments that are in course in the habitat and are maintained by each crew and other 1-week experimental protocols whose topics ranged from geology, biology, botany, engineering, astronomy, and psychology. All the activities were strictly regulated in a schedule defined by the mission control group, which monitored the mission from the outside of the habitat. During the mission, subjects run on “mission time” that started from when they entered the habitat, losing contact with the normal time course. Sleep and wake time were decided by the mission control, and they were progressively reduced during the mission and shifted from normal nocturnal sleeping time creating a shift in normal circadian rhythms that are connected to light and dark cycles. The analog astronauts were also asked to perform some physical activity, in particular during this mission, the crew medical officer established a minimum of 1 h per day. In the habitat, there was a treadmill, an exercise bike and some soft mats for exercises. The subjects were free to choose the kind of activity they preferred, aiming to remain under anaerobic threshold, with a target heart rate of 140–160 bpm. The crew was asked to face emergencies and other unexpected situations during the mission, notified by the mission control in response to real solar activity for example or common emergencies in regular space missions (e.g., air leak, fire, solar storm). Facing simulated emergencies often created highly stressful situations for the crew members that were forced to abandon their working schedule and to rethink the mission plan after spending hours in solving the scenarios. Meals were consumed together, as the crew agreed, to improve social contact between the analog astronauts, following a balanced diet proposed by a nutritionist. Water intake was high, in fact, the crew members were encouraged to drink since in stressing conditions dehydration could be detrimental for performance. No caffeine, alcohol or sweet beverages were allowed, the subjects could drink just water or warm herbal infusions.

During the experiment week a large amount of data were recorded. Every day at 8 a.m., the subjects measured their temperature, body weight, blood pressure, heart rate, arterial pressure, SpO_2_.

**Fig. 1:**
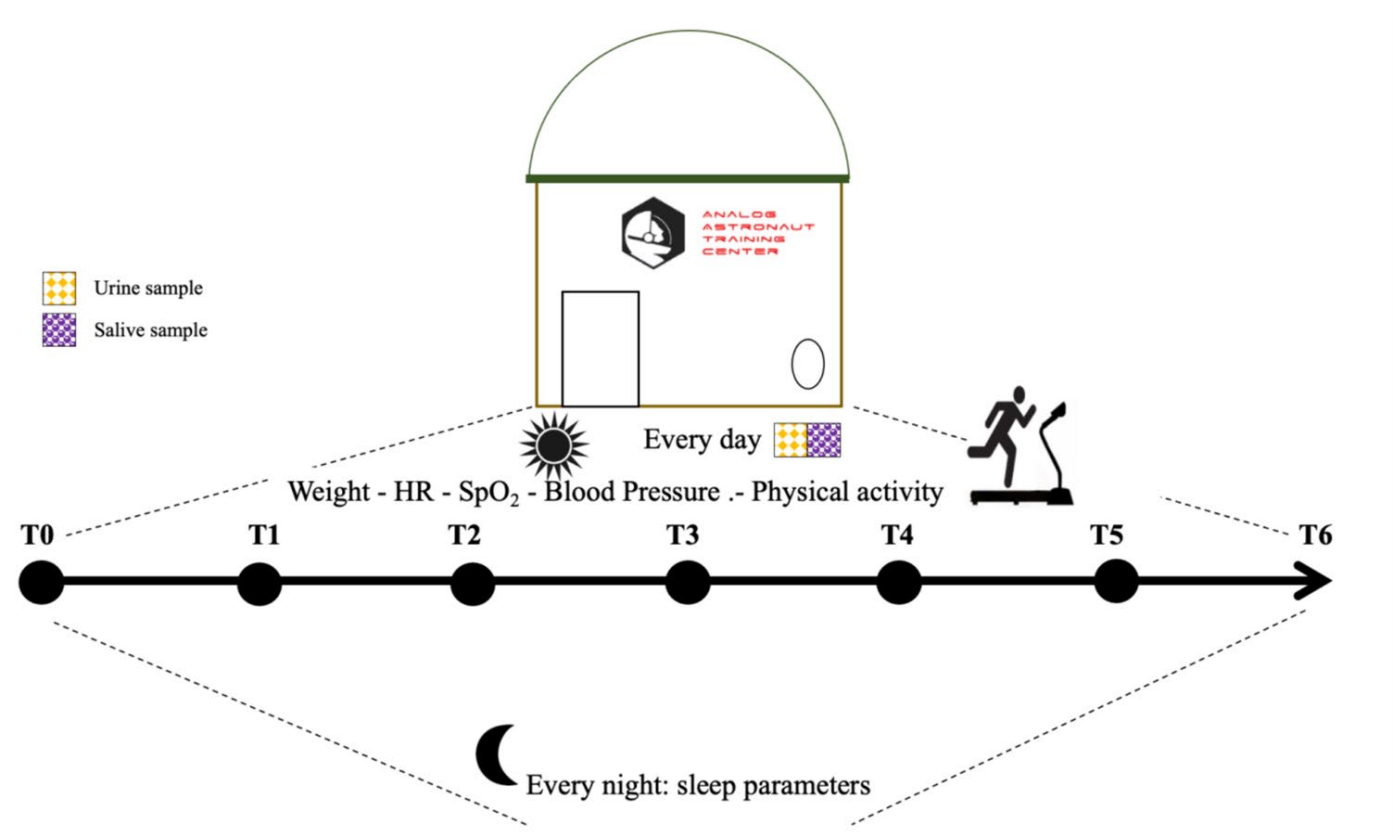
Schema of experimental protocol using saliva and urine samples from T0 to T6 (T0: the first day of the mission and T6 the last day of the mission) in young analog astronauts

Wearable devices (Mi Smart Band 6, Xiaomi, China) were used to monitor daily step count, physical activity, heart rate during physical exercise and at rest, and sleep parameters. Algorithms from the builders app were used in the data analysis since we had no access to raw data from the devices. Each day crew medical officer obtained saliva and urine samples. Saliva samples have been collected immediately after awakening, before breakfast (Fig. 1).

### Ethical considerations

This study was conducted following the Helsinki Declaration and was approved by the Ethical Committee of the Health Authorities of the Province of Padua approved the study (practice number 3843/AO/16). All the volunteers signed an informed consent. The experiment has been conducted in the Analog Astronaut Training Center (AATC) in Poland in October 2021.

### Sample collection

Biofluid was collected every day from T0, the first day of the mission, to T6, the last day of the mission. Saliva samples were collected, about 1 mL, using a Salivette device (Sarstedt, Nümbrecht, Germany), centrifuged at 3000 rpm for 20 min (Mrakic-Sposta et al. 2020; Giacon et al. 2022; Bosco et al. 2023; Brizzolari et al. 2023), aliquoted and stored at − 80 °C until assayed and thawed only once before analysis. Also, urine samples were collected by voluntary voiding in a sterile container, aliquoted and stored at − 20 °C until assayed and thawed only once before analysis.

### Biomarkers assessment in saliva

#### Reactive oxygen species (ROS)

Electron paramagnetic resonance spectroscopy (EPR), 9.3 GHz, X-band, (E-Scan, Bruker Co., MA, USA) was used to detect ROS production, at 37 °C using a Temperature Controller unit (Noxigen Science Transfer & Diagnostics GmbH, Germany), interfaced with the spectrometer. Methods were previously described (Mrakic-Sposta et al. 2020; Giacon et al. 2022; Bosco et al. 2023; Brizzolari et al. 2023), briefly CHM spin probe (1-hydroxy-3-methoxy-carbonyl-2,2,5,5-tetramethylpyrrolidine) was used for ROS production detection, and a stable radical CP· (3-carboxy2,2,5,5-tetramethyl-1-pyrrolidi-nyloxy) was used as an external reference to convert ROS determinations into absolute quantitative values (μmol min^−1^). Spectra acquired were recorded and analyzed using Win EPR software (version 2.11) standardly supplied by Bruker.

#### Total antioxidant capacity (TAC)

The 6-hydroxy-2,5,7,8-tetramethylchroman-2-carboxylic acid (Trolox)-equivalent antioxidant capacity assay, by a widely used kit-based commercial method (No. 709001, Cayman Chemical, Ann Arbor, MI, USA), was used in saliva samples. The method was previously described (Vezzoli et al. 2016).

#### Cortisol

The concentration of free cortisol in the saliva was quantitatively determined through ELISA method according to the protocol of the manufacturer's kit (COR(Cortisol) ELISA Kit; FineTest, Wuhan Fine Biotech Co.) as previously described (Giacon et al. 2022).

#### Leptin and IGF-1

Leptin and IGF-1 levels were measured in saliva by means of enzyme immunoassay (ELISA) kits (cat. No. EH0216; FineTest, Wuhan, China) and (cat. No.EH0165; FineTest, Wuhan, China) respectively. The methods were previously described (Micarelli et al. 2022, 2023).

### Biomarkers assessment in urine

#### 8-isoprostane (8-iso-PGF2α)

Lipid peroxidation was assessed by immunoassay of 8-isoprostane concentration (Cayman Chemical, Ann Arbor, MI, USA) in urine. Samples and standard were read at a wavelength of 512 nm. The results were normalized by the urine creatinine values. The method was previously described (Bosco et al. 2018a, b, 2023; Giacon et al. 2022; Mrakic-Sposta et al. 2019, 2020, 2022; Vezzoli et al. 2023).

#### Interleukin-6 (IL-6)

IL-6 urinary levels were determined by ELISA kit (ThermoFisher Scientific, Waltham, MA, USA), according to the manufacturer’s instructions. The method was previously described (Mrakic-Sposta et al. 2015, 2020; Giacon et al. 2022).

All the samples and standards were read by a microplate reader spectrophotometer (Infinite M200, Tecan Group Ltd., Männedorf, Switzerland). The determinations were assessed in duplicate, and the inter-assay coefficient of variation was in the range indicated by the manufacturer.

#### Creatinine, neopterin and uric acid

Urinary creatinine, neopterin, and uric acid concentrations were measured by isocratic high-pressure liquid chromatography (HPLC) method, as previously described (Dellanoce et al. 2014; Vezzoli et al. 2016) over the range of 0.125–1 μmol/L, 0.625–20 mmol/L, and 1.25–10 mmol/L for neopterin, uric acid, and creatinine levels, respectively. Inter-assay and intra-assay coefficients of variation were < 5%. Methods were previously described (Glantzounis et al. 2005; Mrakic-Sposta et al. 2019; Giacon et al. 2022).

### Physical exercise

Daily physical exercise was strongly encouraged. Subjects could freely choose to either run on a treadmill (Urbogym V620MS, Urbogym, Poland) or cycling on an exercise bike (SportPlus sp rb 950 i.e., sportplus, Hamburg, Germany). Normal fitness exercises such as push-ups and crunches and muscles stretching were recommended before and after the main exercise. Distance and duration of the effort were controlled both on the fitness device itself and through the wearable device (Mi Smart Band 6, Xiaomi, China) which also monitored heart rate, daily step count and esteemed caloric expenditure.

### Sleep

Sleep monitoring was obtained through both a normal chronometer to monitor the duration of sleep and to further investigate sleep phases and sleep duration we utilized data from the wearable devices (Mi Smart Band 6, Xiaomi, China). Sleep quality was self-reported by the subjects following a 1 (worst) to 10 (best) Visual Analogue Scale (VAS). Sleep duration was determined by mission control, outside of the habitat, and it was shifted from normal light cycle. Sleep debt increased during the week. The crew slept in a small room, in bunk beds, without switching off artificial light.

## Data collection and analysis

Statistical analysis was performed with SPSS Statistics (SPSS version 25- IBM), GraphPad Prism package (GraphPad Prism 10.0.1, GraphPad So ware Inc., San Diego, CA) for Mac and Python v3.12.2, using the pingouin open-source statistical package (Vallat 2018). Kolmogorov–Smirnov testing was utilized to determine the distribution of each data set.

To verify that the residuals are normally distributed**,** scipi.stats.shapiro_wilk_test method was used. Data were compared by ANOVA repeated measures followed by Dunn’s multiple comparison test to further check the groups’ significance. Cohen with 95% CI was used for calculating the size effect.

We used the repeated measures correlation, rm_corr measure, a statistical technique for determining the common within-individual association for paired measures assessed on two or more occasions for multiple individuals (Bakdash and Marusich 2017).

All data are presented as mean ± S.D., and significance was determined at *p* < 0.05. Change Δ% estimation (((post value – pre value)/pre-value) × 100) is also reported in the text.

## Results

Physiological parameters obtained at the different time-points during the sojourn in the habitat are reported in Table 2.

**Table 2:** Physiological parameters collected from the subjects from T0 to T6, every day at 8 a.m

Physiological parameters	T0	T1	T2	T3	T4	T5	T6
Temperature (°C)	36.4 ± 5	36.6 ± 0.4	36.8 ± 0.2	36.5 ± 0.4	36.4 ± 0.3	36.5 ± 0.3	36.5 ± 0.3
SBP (mmHg)	125.6 ± 8.7	118.6 ± 7.2	110.2 ± 9.1	104.6 ± 9.5	115.6 ± 8.9	115.8 ± 8.3	120.6 ± 7.4
DBP (mmHg)	82.6 ± 3.1	80.0 ± 3.1	79.6 ± 6.6	64.4 ± 5.7	74.6 ± 10.3	78.4 ± 5.1	79.4 ± 4.1
HR (bpm)	82.2 ± 19.4	77.2 ± 18.1	73.0 ± 13.8	69.0 ± 12.8	73.8 ± 14.5	77.4 ± 14.1	80.4 ± 15.5
SpO_2_ (%)	98.2 ± 1.1	97.8 ± 1.8	97.4 ± 1.3	97.6 ± 2.6	98.6 ± 0.5	98.2 ± 0.4	97.2 ± 1.1

Data are expressed as mean ± SD

*SBP* systolic blood pressure, *DBP* diastolic blood pressure, *HR* heart rate

## Oxy-inflammation

**Fig. 2:**
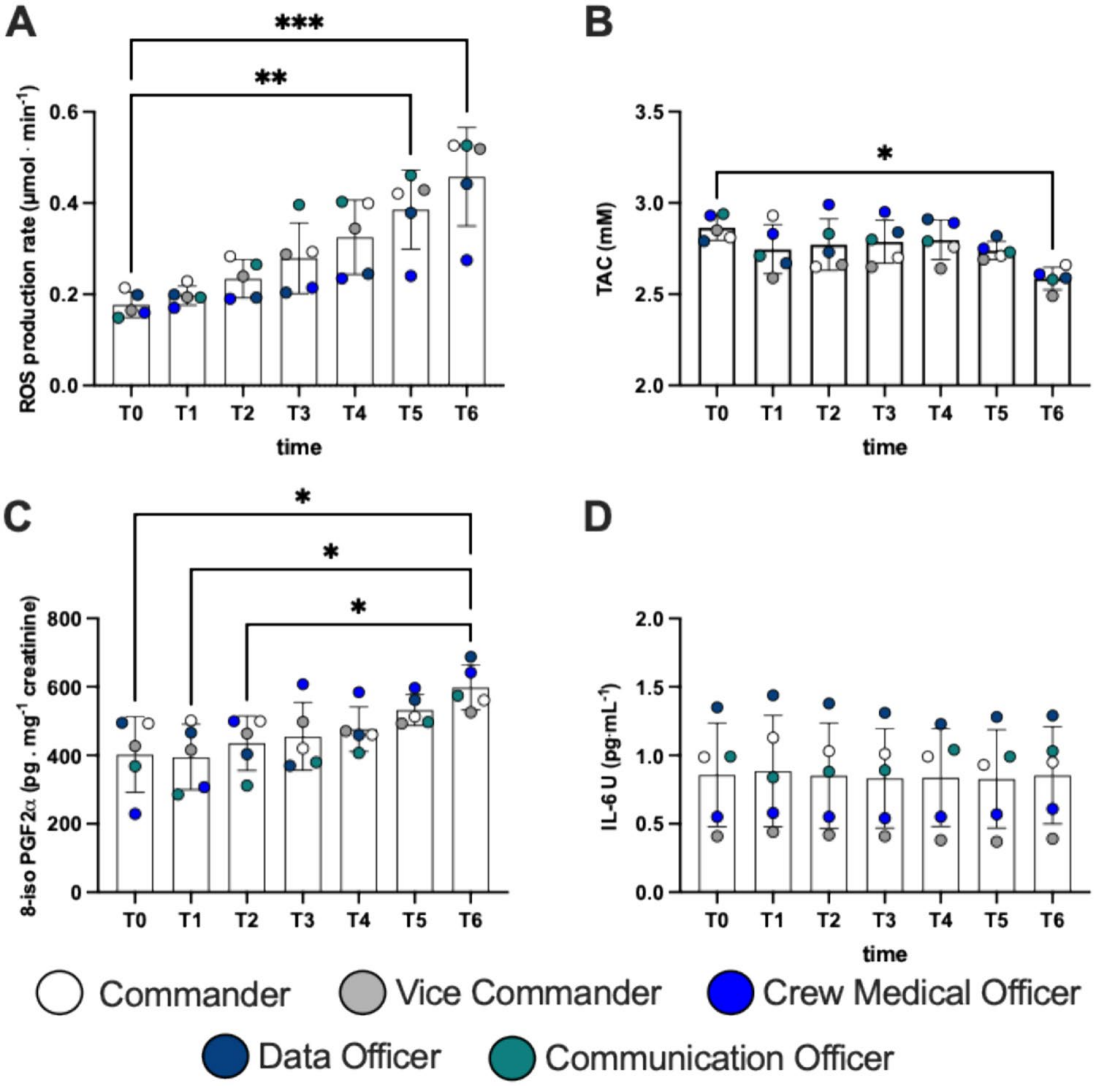
Histogram panel plots of the oxidative stress biomarkers. Time course of salivary **A** reactive oxygen species (ROS) production, **B** antioxidant capacity (TAC) and urinary **C** lipid peroxidation (8-iso), **D** interleukin 6 (IL-6) levels. Data are expressed as mean ± SD. Statistically significant differences comparisons are displayed as: *p < 0.05; **p < 0.01; ***p < 0.001

The concentration values of oxidative stress biomarkers obtained during the mission from T0 and T6 in the examined subjects are displayed in Fig. 2.

We found a significant increase in ROS production at T5 and T6 vs T0: + 114%, *d*Cohen = 3.26; and + 158% *d*Cohen = 3.55 (Fig. 2A). TAC slightly decreases at T6 compared to T0: -10%, *d*Cohen = 5.75 (Fig. 2B), while the marker of lipid peroxidation increased at T6 vs T0, T1 and T2: + 49%, *d*Cohen = 3.77; + 51%, *d*Cohen = 4.09; and + 37%, *d*Cohen = 0.66 respectively (Fig. 2C). Urinary IL-6 did not show any statistical difference (Fig. 2D).

## Hormones

**Fig. 3:**
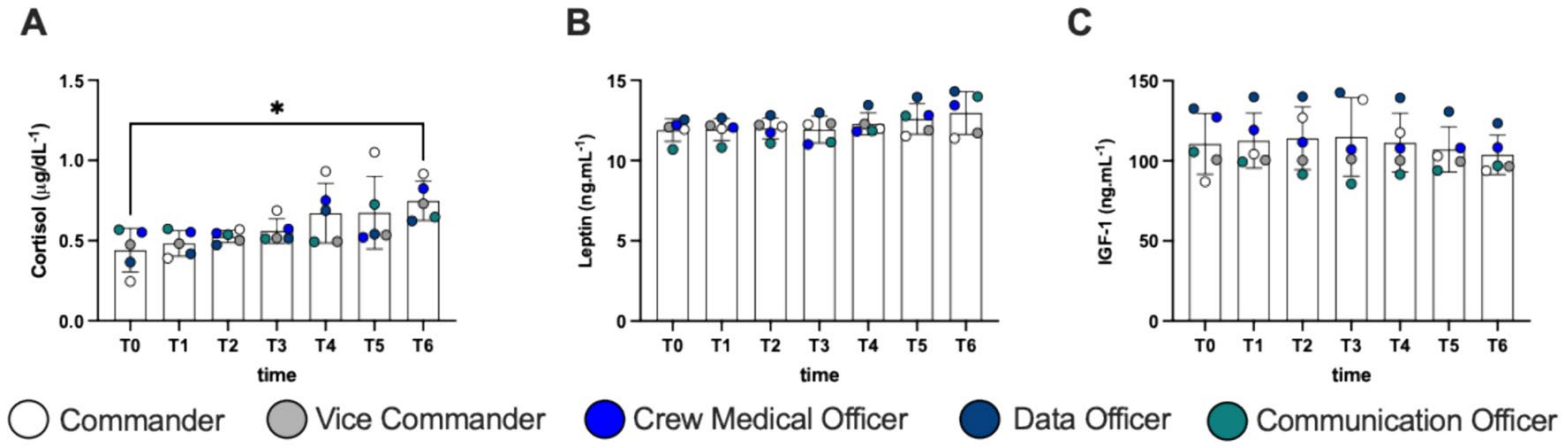
Histogram panel plots of hormones. Time course of **A** cortisol, **B** leptin, and **C** IGF-1. Data are expressed as mean ± SD. Statistically significant differences comparison are displayed as: *p < 0.05

A significant increase in salivary cortisol levels was measured at T6 vs T0 + 70%, *d*Cohen = 2.39, (Fig. 3A). Leptin and IGF-1 did not show statistical differences (Fig. 3B, C).

## Renal function

**Fig. 4:**
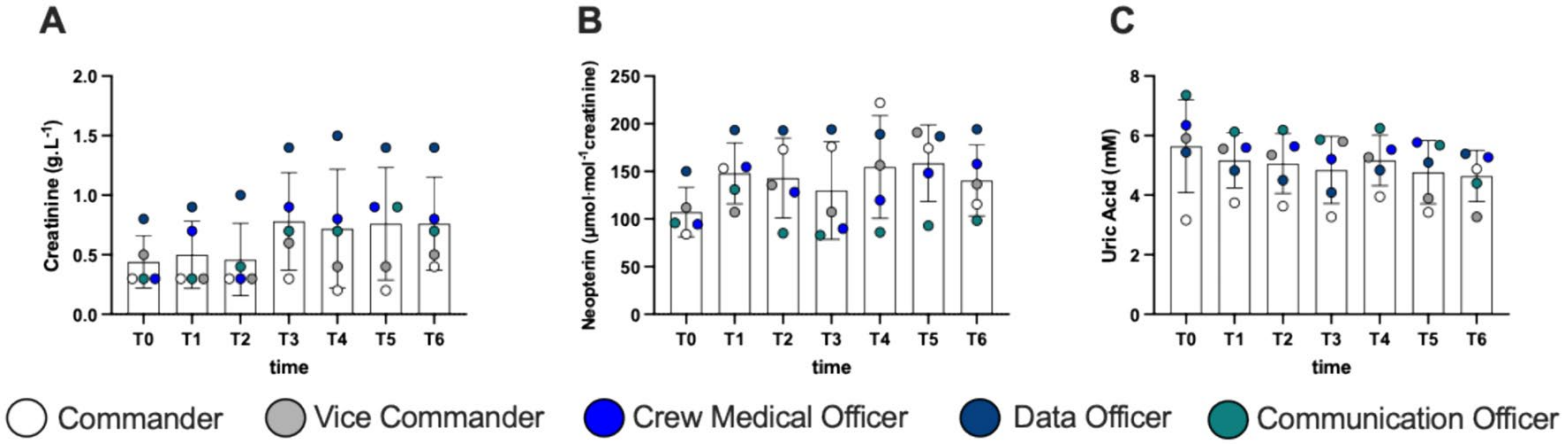
Histogram panel plots of renal functions biomarkers. Time course of **A** creatinine, **B** neopterin, and **C** uric acid. Data are expressed as mean ± SD

No significant differences were found in biomarker of renal function obtained during the mission (Fig. 4).

## Exercise

**Fig. 5:**
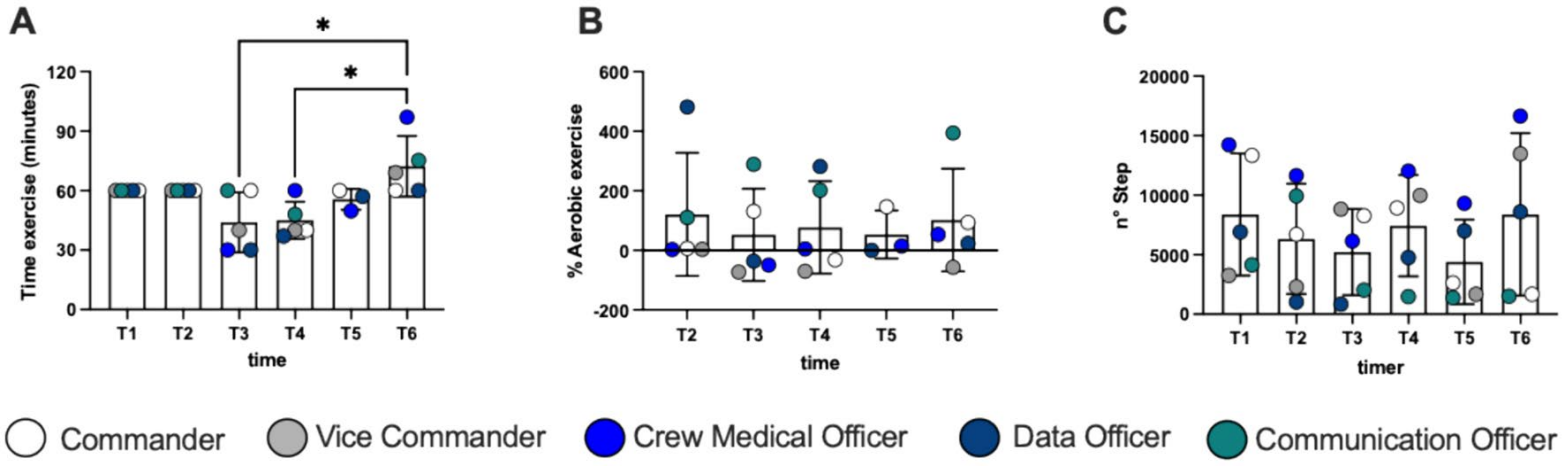
Histogram panel plots of time of exercise (**A**), % Aerobic exercise (**B**) and number of steps (**C**). Data are expressed as mean ± SD

We found a significant increase in time of exercise during the mission (Fig. 5A) at T3 and T4 vs T6: + 64%, *d*Cohen = 1.85; and + 61% *d*Cohen = 2.15, respectively. No significant differences were found in different type of exercise (Fig. 5B, C). Three of the subjects recorded their daily step-count also for 1 week after the mission. On average, they reported 5926 ± 2619 steps per day which is no different from the values obtained inside the habitat.

## Sleep

**Fig. 6:**
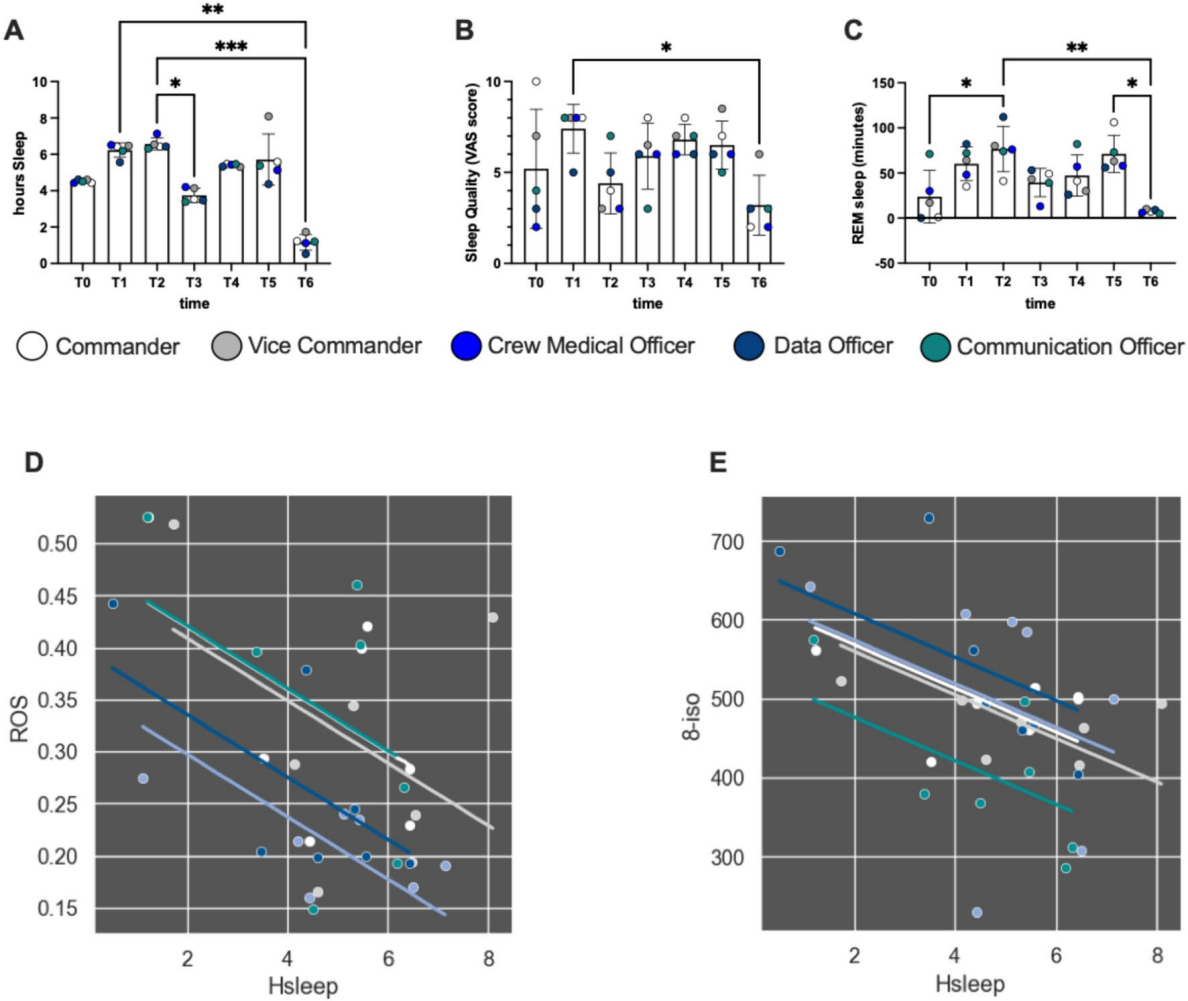
Histogram panel plots of hours of sleep/day (**A**), sleep quality (**B**) and REM phase of sleep (**C**). Data are expressed as mean ± SD. Plots partial correlation, between hours of sleep/day and the ROS production rate (**D**), and 8-isoprostane (**E**), (recorded in every subject at every day) are reported in each panel. Statistically significant differences are displayed as: *p < 0.05; **p < 0.01; ***p < 0.001

A significant decrease in hours of sleep/day, sleep quality and REM phase of sleep was recorded. In detail: hours of sleep/day decreased at T6 vs T1 − 81%, *d*Cohen = 12.35; T2 vs T3 − 43%, *d*Cohen = 7.33; T2 vs T6 − 82%, *d*Cohen = 14.27 (Fig. 6A). The sleep quality (VAS) decreased at T6 vs T1 − 57%, *d*Cohen = 0.288 (Fig. 6B). Finally, the REM phase of sleep calculated in minutes from wearable devices data, changed during the nights of the mission: at T2 vs T0 + 69%, *d*Cohen = 1.93; T2 vs T6 − 90%, *d*Cohen = 3.87; at T5 vs T6 90%, *d*Cohen = 4.37 (Fig. 6C).

Significant relationships were found among hours of sleep/day and ROS production (*r* = − 0.520; *p* = 0.003; Fig. 6D), and 8-iso concentrations (*r* = − 0.505; *p* = 0.004; Fig. 6E).

## Discussion

In this work, we studied the effects of a simulated space mission on five young healthy subjects and investigated the responses of the crew to confinement, alteration of sleep and high workload, analyzing different biomarkers ranging from oxidative stress, inflammation, and hormonal response.

### Oxy-inflammation responses during analog mission

An unbalance in oxidative profile has rapidly developed from the early moments of the mission. In fact, a progressive increase in ROS production has been detected (Fig. 2A), with a significant decrease in antioxidant capacity at the end of the week (Fig. 2B).

Our previous experiences have been focused on studies in extreme conditions: saturation divers (Mrakic-Sposta et al. 2020), offshore ocean sailing (Giacon et al. 2022) and Antarctic Plateau winter-over (Mrakic-Sposta et al. 2022). All studies focused on a small population of subjects that performed for a medium-long term period some physically demanding activities under highly stressful environmental conditions, isolated and confined. It is really interesting to confirm such a pattern in analog astronaut missions too, both because we can assume that other confined and stressful environments could serve as surrogates to make inferences on analog astronauts, and because in this case, compared with underwater, sailing, and/or high altitude, the environment was less harsh to the subjects but nevertheless it determined high levels of oxidative stress.

The unbalance between ROS production and TAC led to the resulting lipid peroxidation (Fig. 2C). When the concentration of ROS exceeds a certain limit, the unpaired electrons react with neighboring molecules, inducing new free radical generation (Sies and Jones 2020). This process can be free radical chain reactions, and subsequently lead to oxidative damage of proteins, lipids, amino-acids, DNA, changing and often impairing the function of these macromolecules (Pizzino et al. 2017; Juan et al. 2021).

Despite this, there were no significant changes in the inflammatory status that we have been able to measure; IL-6, a generic marker of inflammation showed no significant changes. Furthermore, oxidative stress and inflammation are two phenomena that are directly involved in the aging process (Bevere et al. 2022) and especially in pathology status (Bosco et al. 2018b; Moretti et al. 2018; Hajam et al. 2022). Our sample consisted of a team of healthy and young subjects and probably for this reason, even given the limited confinement time, the biological system was not deeply affected by this exposition.

### Hormonal responses during analog mission

Hormonal response to stress usually determines an increase in endogenous glucocorticoids and mineralocorticoids production (Whirledge and Cidlowski 2010). Under circumstances such as the above described, subjects are under extremely stressful conditions, therefore we decided to obtain a cortisol profile during the mission. We documented a steady increase in cortisol levels (+ 40%), measured at awakening, during the 7 days of mission (Fig. 3A). Cortisol curve flattening during sustained stressing conditions is often reported. In fact, lack of morning peaks in cortisol secretion represents a dysregulation in glucocorticoids homeostasis, in response to an altered circadian rhythm or other stressors. For example, sailors who face long lasting ocean passages and are exposed to both physical and environmental stressors, tend to sleep in shifts and completely lose the light and darkness cycle, and sustained higher levels of cortisol are reported (Gunnarsson et al. 2004). Moreover, disruption of circadian rhythm can interfere with daily activities (Lightman et al. 2021). During the MARS500 long term isolation study, higher levels of cortisol derived just from isolation (Jacubowski et al. 2015). In terms of sex differences, during an analog mission in Antarctica, female subjects had higher levels of cortisol compared with males (Strewe et al. 2019).

Despite individual variations, no significant changes in leptin and IGF-1 concentrations were reported (mean range variation + 9% in leptin and + 6% in IGF-1). However, these variations can lead to an alteration of physical stress, as reported by Bouillon-Minois et al. (2021). During the mission high importance has been given to nutrition and hydration. Subjects were recommended to drink abundant water. Moreover, no caffeine or theine were admitted to the habitat. Biomarkers of renal function: creatinine, neopterin and uric acid showed no changes.

### Physical activity, sleeping and monitoring during analog mission

Subjects were required to practice about one hour of exercise per day. During some days this has not been possible due to emergencies simulations (e.g. T3) or tight working schedule, but in general an optimal adherence to exercise has been recorded. This surely impacted on stress perception and on physical condition. In fact, confinement in tight spaces and desk work is highly detrimental to physical performance, in our daily life (Tai et al. 2022), in extreme earth-based situations such as sailing and submarines (Leach 2016) but most importantly in space (Koschate et al. 2021).

During the mission, even though there has been a high interpersonal variability, we recorded an increase in physical activity duration (Fig. 5A) meaning that subjects considered it increasingly important during the days, and devoted a larger portion of their free time to physical activity.

Few data are present in the literature on the relationship between oxidative stress and sleep in humans. Despite humans sleeping nearly a third of their lives, the functions of this physiological state remain unknown (Hill et al. 2018). Prolonged episodes of sleep deprivation in human cause an increased need to sleep, increased metabolic (Nedeltcheva and Scheer 2014) and cardiovascular disorders (Cappuccio et al. 2011), cognitive impairment/psychiatric disorders (Alhola and Polo-Kantola 2007; Baglioni et al. 2011), inflammation (Faraut et al. 2012; Ferrie et al. 2013) and in severe cases lead to death (Hublin et al. 2007; Gallicchio and Kalesan 2009). Already in 1944, Reimund (Reimund 1944), in a theoretical hypothesis, proposed that reactive oxygen species (ROS) accumulate in neurons during the wake state and that sleep allows the clearance of ROS in the brain. Indeed, these are chemically reactive by-products of metabolism which, when not properly neutralized, can damage lipids, proteins, DNA and can lead to cell death. Despite that, the relationship between chronic sleep restriction and oxidative stress has not been thoroughly investigated, despite the physiological relevance of chronic sleep restriction widespread in modern society (Liu et al. 2016). Our data show a relationship between hours of sleep and selected oxidative stress biomarker, as shown in Fig. 6D, E. As the hours of sleep decrease, the production of ROS increases, with a consequent increase in the damage to the membrane lipids.

The necessity of remotely monitoring the health status of aerospace crews has contributed to develop many telemedicine and telemonitoring systems and, as for many technological cutting-edge technologies, they have been subsequently employed for earth applications (Nicogossian et al. 2001). Monitoring physical parameters and performance is gaining popularity due to the accessibility and accuracy of wearable devices. In some cases, the advantages that they offer in terms of ease of data collection, visualization, and interpretation are favorable in their scientific use. Even though not all of them are intended and validated for medical use, they provide discretely precise information for a descriptive purpose, that often differ by small percentage from data obtained with dedicated instruments (Izmailova et al. 2018). Different models can be used to monitor sleep quality and quantity (Chinoy et al. 2021), energy expenditure (Düking et al. 2020), saturimetry (Lauterbach et al. 2020) and heart rate (Navalta et al. 2020). Data obtained from the wearable devices we used (Xiaomi, Mi smart Band 6, Xiaomi, China) have been adopted mostly for the subjects to be able to gain consciousness of their physical and health status and to avoid inactivity, with optimal efficacy as reported in Figs. 5 and 6. Data from these devices must be cautiously evaluated, since the devices are not validated for medical use. Nevertheless, uniform monitoring was obtained throughout the crew with these simple and user-friendly devices and we believe it has been really useful. However, data show a deterioration in the quality of sleep and we can therefore hypothesize an initial alteration of the circadian rhythms with consequent desynchronization that may occur as a consequence, as described by Schaefer et al. (1979).

## Limitations

This pilot study’s reliability is limited by the small number of subjects included in the study, their male gender prevalence and limited age range. Moreover, a large amount of generic data was collected, but more detailed investigation topics would be auspicable to acquire more relevant results. Data acquired with commonly available and often not scientifically validated wearable devices should be interpreted with particular care. We do not think they should be discarded, since precision of the data collection should be commensurate with the needed precision and the importance of them on the final result. Even considering a fair amount of error, we think that the final result is not affected in terms of its descriptive potency. Moreover, an important aspect of using these simple commercially available devices is that they reflect the truth of many situations in which they are commonly employed. We live daily with devices that measure our step count, calories and sleep duration, and we still rely on them for our purposes. Utilizing scientifically validated devices would be ideal for a prosecution of the study, but their limited availability often does not reflect the reality of their applicational environments. Subjects should also be studied for longer periods of time, including some reference periods before and after the mission phase, to improve data reliability. In this case, we managed to obtain data from only three subjects after the mission due to lacking of reporting. Keeping in mind that individual differences in terms of stress response and adaptation could influence the results of a study, in particular in small crews as for astronauts, for example, (Bartone et al. 2018) we think we have given an interesting perspective on stress response in analog astronauts.

## Conclusion

The analysis suggested that the crew have suffered from really high-stress levels, with an increase in levels of cortisol, ROS production and related oxidative damage to lipids, and parallel decrease in antioxidant defense in just a few days; furthermore, sleep quality significantly deteriorated, hours of sleep decreased, and REM sleep was also affected. The rapid installation of good habits, such as regular physical activity and adequate nutrition and hydration, hampered the loss of physical performance, always keeping the functional parameters good and not influencing the inflammatory states throughout the stay in the habitat. This study deepened our understanding of challenges astronauts might face during extended space travel, shedding light on possible health decline mechanisms in space. In sum, the study advanced our knowledge of physiological changes during prolonged stressful conditions comparable to space missions, offering potential strategies to address health risks and enhance future space exploration opportunities.

## Data Availability

All raw data that were presented or mentioned in the manuscript are available from the corresponding authors on request.

## Abbreviations

BMI: Body Mass Index
EPR: Electron paramagnetic resonance
HPLC: High-performance liquid chromatography
IL-6: Interleukin-6
IGF-1: Insulin-like growth factor
OxS: Oxidative stress
ROS: Reactive oxygen species
REM: Rapid eye movement
TAC: Total antioxidant capacity
8-iso-PGF2α: 8-Isoprostane
